# Newborn Screening for Cystic Fibrosis: Over the Hump, Still Need to Fine-Tune It

**DOI:** 10.3390/ijns6030057

**Published:** 2020-07-09

**Authors:** Carlo Castellani

**Affiliations:** Cystic Fibrosis Center, IRCCS Istituto Giannina Gaslini, 16147 Genoa, Italy; carlocastellani@gaslini.org

Today, newborn screening (NBS) is considered an essential component in the standards of care for cystic fibrosis (CF) [1] and, to cite a well-known paper, “a basic human right” [2]. This has not always been the case and, in a not too remote past, the appropriateness of screening neonates for CF was much debated. In those days, NBS had been implemented in very few areas, and was more often a research project than an established health program. Decision-makers were waiting for proof that early diagnosis was an opportunity to modify the natural history of CF. That sort of evidence was not easy to collect for a disease characterized by wide genotype and phenotype diversity and a long term clinical evolution, and the very few randomized controlled trials struggled to prove the point [3,4]. Over time, direct and circumstantial evidence in favour of the benefits of CF NBS accumulated [5] and its practice progressively extended to most countries with predominantly Caucasian populations. A further acceleration came from the emergence of small molecules targeting the defective CF transmembrane regulator (CFTR) protein. These compounds, although only partially rescuing CFTR function and not yet available to all patients or licensed for use in the first months of life, might prevent or greatly delay the development of disease manifestations if started at the youngest possible age.

Currently, the vast majority of newborns in North America and Europe, and a growing number in South America, are screened for CF. The expansion phase of CF NBS has probably reached its summit and is gradually slowing down. Nevertheless, it remains crucial and urgent to support the implementation of NBS in countries where CF shows a significant prevalence, that can capitalize on the competence accumulated elsewhere and avoid the errors made by those who preceded them.

It is also critical to make the actual screening strategies as effective and as efficient as possible. Guidelines are available [1,6] and provide direction, but advice can be challenging to implement in distinct genetic, logistic and strategic environments. There is no model that fits all the variables that characterize different areas, and each protocol has to be customized for local needs. Sharing expertise and learning from others’ experience may help to tune up the many components of each screening strategy and, on a personal level, to improve the daily practice of lab workers, CF doctors and nurses.

The articles in this issue of the *International Journal of Neonatal Screening* offer a state-of-the-art scrutiny of several aspects of CF NBS and contribute to the debate on some old but still burning questions. Some of them are connected with the inclusion of molecular genetics in CF NBS, now used in most protocols for its potential to improve specificity and the timing of the screening procedures. Technological improvements have made it possible and affordable to tailor mutation panels to local requirements but have also offered the option to move to non-mutation-specific analysis. Next generation sequencing allows for the fast identification of all exome variations, with a sensitivity far superior to any pre-set mutation kit. This does constitute an asset in populations with extreme genetic variability, but it may uncover information whose clinical significance is difficult or even impossible to interpret.

CF screening positive, inconclusive diagnosis (CFSPID), also known as CFTR related metabolic syndrome (CRMS), in infants may be detected by NBS strategies that do not include genetic analysis, but many more are found if DNA in IRT-positive samples is sequenced. We are now witnessing a situation somewhat similar to that already experienced with the identification of carriers through CF NBS. This was and is still seen with favour by some, who consider it an opportunity to explore the extended family of the carrier neonate and find couples at high risk of having children with CF, whereas most consider it an undesirable effect of the screening procedure. Similarly, the identification of CFSPID/CRMS infants may be regarded as the occasion for monitoring children who might, over time evolve CF, or as a distressing intrusion in the life of parents whose child may develop late and mild, or even no symptoms at all. None of these considerations can be dismissed as incorrect, but if we agree that the purpose of CF NBS is the early finding of infants with a severe disease and, thereby, to be able to offer prompt treatment, carrier and CFSPID/CRMS children are probably to be considered more an unwanted consequence than a collateral benefit of CF NBS. Concerns about the inclusion of molecular analysis in CF NBS have driven the search for non-genetic assays that could compensate for the limited specificity of IRT. So far, the only option appears to be the pancreatitis associated protein (PAP), which cannot substitute IRT but rather complements it in elaborate screening algorithms.

CF NBS has reached a mature stage of its development and is widely considered an indispensable part of CF care. The debate has now shifted from usefulness to optimization and focused on the containment and management of collateral outcomes, reliable data collection in specific registries and quality monitoring. It is important to keep the dialogue alive among stakeholders, and in this regard this Special Issue is a valuable and timely resource.
